# Good Guy or Bad Guy? The Duality of Wild-Type p53 in Hormone-Dependent Breast Cancer Origin, Treatment, and Recurrence

**DOI:** 10.3390/cancers10060172

**Published:** 2018-05-31

**Authors:** Eileen M. McGowan, Yiguang Lin, Diana Hatoum

**Affiliations:** 1 Central Laboratory, The First Affiliated Hospital of Guangdong Pharmaceutical University, Guangzhou 510080, China; 2 School of Life Sciences, University of Technology Sydney, Sydney 2007, Australia; yiguang.lin@uts.edu.au (Y.L.); diana.hatoum@student.uts.edu.au (D.H.)

**Keywords:** breast cancer origin, p53 tumor suppressor, latency, treatment, estrogen receptor, pregnancy

## Abstract

“*Lactation is at one point perilously near becoming a cancerous process if it is at all arrested*”, *Beatson*, *1896.* Most breast cancers arise from the milk-producing cells that are characterized by aberrant cellular, molecular, and epigenetic translation. By understanding the underlying molecular disruptions leading to the origin of cancer, we might be able to design novel strategies for more efficacious treatments or, ambitiously, divert the cancerous process. It is an established reality that full-term pregnancy in a young woman provides a lifetime reduction in breast cancer risk, whereas delay in full-term pregnancy increases short-term breast cancer risk and the probability of latent breast cancer development. Hormonal activation of the p53 protein (encode by the *TP53* gene) in the mammary gland at a critical time in pregnancy has been identified as one of the most important determinants of whether the mammary gland develops latent breast cancer. This review discusses what is known about the protective influence of female hormones in young parous women, with a specific focus on the opportune role of wild-type p53 reprogramming in mammary cell differentiation. The importance of p53 as a protector or perpetrator in hormone-dependent breast cancer, resistance to treatment, and recurrence is also explored.

## 1. Introduction

### 1.1. Overview of Breast Cancer and Pregnancy 

Breast cancer is endemic worldwide, with a cumulative lifetime risk of 1:8 by age 70 [1,2,3,4]. About 80% of all breast cancers are late-onset, arising in post-menopausal women, and are mainly estrogen receptor alpha (ERα)-positive (ERα+) and p53 wild-type (p53wt) [5]. The association between female hormone regulation and breast cancer has been known for over a century, since the first pioneering operations by Sir George Beatson in 1895 where he showed that removal of the ovaries, the source of estrogen and progesterone hormones, prevented breast cancer recurrence [6]. The specific mechanisms of the origin of breast cancer are still under intense investigation to date, over 120 years later. The understanding of the mechanisms underlying the origin of breast cancer has important therapeutic implications and is relevant to both prevention and treatment. It is undisputable that fluctuations in female hormones throughout a woman’s lifetime influence the risk of late-onset breast cancer, with substantial evidence that the timing of the first pregnancy has long-term impact on breast cancer risk. In today’s society, there is a global trend for women to have fewer pregnancies at later ages, and this trend is associated with an increased incidence of breast cancer [1,3,4,7,8,9]. Therefore, understanding the molecular changes associated with the reduced breast cancer risk that is observed in young parous females is imperative for the development of effective approaches to reduce breast cancer incidence and to improve treatment outcomes.

### 1.2. p53 in the Differentiation of the Breast and in Breast Cancer

There is a very strong association between the function of the p53 tumor suppressor (encoded by *TP53* gene) and female hormone regulation during the differentiation of the mammary gland in early and late pregnancies, which influences a woman’s susceptibility to breast cancer later in life [10,11,12,13]. This leads to the broader question: is the almost total breast cancer refractoriness in young parous or multiparous women due, in part, to the influence of p53 on the differentiation of the breast in a “critical time window”? In this article, to partly answer this question, we look into early studies of p53 as a key regulator of normal breast cell physiology and its role in hormone-dependent breast pathophysiology. The second most pressing question we address is the role of p53 as a protector or perpetrator in hormone-dependent breast cancer recurrence and resistance to treatment. 

This article will review what is known about the protective influence of female hormones in young parous women on the basis of historical and contemporary findings in published studies. We will then focus on the duality of wild-type p53, as a good guy as well as a bad guy, in the origin of breast cancer, as a protector and/or perpetrator. How this may impact on hormone-dependent breast cancer treatment and recurrence will also be discussed. 

## 2. Female Hormones, Pregnancy, and Prevention/Promotion of Breast Cancer

The conundrum that female hormones have a dual effect on breast cancer risk, both protective and causative, has been at the forefront of discussions on the etiology of breast cancer origin for the last century. Seemingly contradictory evidence has demonstrated that administration of estrogen and progesterone, in a ‘window in time’ in young-aged pregnancies may provide a life-long protection against breast cancer [14,15,16,17,18]. Alternatively, the female hormones estrogen and progesterone are well-known mitogens in breast cancer progression [19,20]. The surgery performed by Beatson in 1895 [21] was heralded as the first hormonal therapy for breast cancer. Dating back over 50 years, first-line therapies for hormone-dependent cancers are antiestrogen-based treatments: tamoxifen, blocking ERα, and aromatase inhibitors, namely, anastrozole and letrozole, blocking estrogen production [22,23,24]. However, the very hormones that we target for breast cancer therapy have also been demonstrated to be the very reason why early-aged full-term pregnancy provides long-term protection against tumor formation.

The number of children and the age of the first pregnancy determines a woman’s short-term and long-term risk of breast cancer. It is not surprising that with the flux of hormones during pregnancy there is an enhanced short-term risk of breast cancer compared with nulliparous women, and this imminent risk increases with increasing age at first birth [25,26,27]. This transitory increased risk in young female pregnancies is offset by multiple births and also brings with it a long-term benefit of reduced latent breast cancer. However, the latent protective effect is not observed in women who have children over the age of 35 [25,26,27]. The golden question is: what transformations occur in the developing lactating breast that provide long-term protection against latent breast cancer without increasing the short-term risk? This complex question is partly answered by molecular studies in rodents and is discussed in more detail in Section 4.

Fundamentally, breast cells divide rapidly during pregnancy, so any genetic alterations occurring in these cells at the time of proliferation will be copied. Genetic alterations (multiple DNA mutations) and post-genetic alterations (i.e., methylation and chromatin re-modeling) during pregnancy are strongly implicated in breast cancer development and protection [28].

## 3. Differentiation in the Mammary Gland during Pregnancy and the Origin of Breast Cancer

Late-onset breast cancers are more likely to originate from ERα-positive luminal epithelial cells, or milk-producing cells, which have been exposed to years of fluctuating hormonal changes from puberty to pregnancy and menopause [29]. As mentioned in Section 2, the best-recognized risk factors for breast cancer are the hormonal changes during pregnancy.

There is irrefutable, reproducible evidence from human studies and in rodent models that full-term and multiple pregnancies have a lifetime protective effect against breast cancer [30,31,32]. Rodent models proved to be well-suited for studying the early development of the mammary gland during puberty and gestation and the effect of a carcinogenic insult in the different stages of breast development [33,34,35,36,37]. In rats, breast tumors can be induced by intravenous or subcutaneous *N*-methylnitrosurea (MNU) or oral 7,12-dimethylbenz(a) anthracene (DMBA), with a near 100% latency period between 8 and 12 weeks [38]. Pioneering experimental studies in mice and rats (from 1973) provided some insight into the morphological changes occurring in the mammary gland of nulliparous women compared to parous women: undifferentiated or partially differentiated glands in young females were more susceptible to neoplastic transformation upon carcinogenic insult (reviewed in [19,39]). During puberty, under hormonal regulation, terminal lobular units of the breast (lobules type 1) progressively develop and differentiate into type 2 and type 3 lobules [40]. In pregnancy, also under the influence of female hormones, these lobules enlarge progressively to a fully differentiated secretory lobule (type 4). The mammary gland regresses to a different morphology to that of a virgin rat post-involution, and its cells remain more differentiated post-lactation [39,40]. An interesting study by Russo and Russo in 1980, looking at the influence of differentiation on cell cycle kinetics in the parous and non-parous breast of young and older rats [41], found that there was some disparity in cell cycle progression, whereby the cells of non-parous rats were more proliferative. On the basis of these studies, it was suggested that the protective effect of pregnancy was partly due to modifications of the G1 or G0 phases of the cell cycle in such a way to make breast cells refractory to transformation, speculating that the prolonged resting stage of cells in G1 phase allowed for more efficient DNA repair. Later studies showed that DNA repair was more efficient in mammary epithelial cells of parous animals (compared to virgins), and DNA was less susceptible to bind DMBA, a commonly used carcinogen shown to induce tumor formation in rats and mice [38,42,43]. An interesting suggestion from these studies was that the limited proliferation capacity in parous rats was due to commitment of function in pregnancy-induced differentiation. This was associated with specific cell cycle modifications in the G0–G1 phases. Phenotypic studies of histological sections of human breast tissue revealed distinct alterations in the structure of the nuclear compartment of non-parous versus parous breast cells, indicating a shift from non-condensed euchromatin to a more condensed heterochromatin structure, respectively [44]. This observation was supported by transcriptomic studies showing the upregulation of genes involved in chromatin reorganization and epigenetic changes induced during pregnancy [44]. Chromatin remodeling and epigenetic changes were also demonstrated by increased histone methylation, an important posttranscriptional process in gene silencing. As evidenced by these studies, there is a clearly defined stepwise coordinated set of cellular metabolic reprogramming, from undifferentiated to pre-secretory differentiation, to functional differentiation, i.e., lactating breast.

From early studies in rodents, it was unclear whether completion of full differentiation per se (in the absence of estrogen and progesterone) reduced breast cancer risk. A few studies tested whether induction of full differentiation of the mammary gland was indeed the basis of protection against carcinogenicity [10,14,45]. Perphenazine (PPZ) is a dopamine receptor inhibitor known to chemically differentiate mammary glands by inducing prolactin release from the anterior pituitary gland. Using PPZ, fully differentiated mammary glands were actually found to be susceptible to carcinogens [10,14,45], seemingly in contradiction to previous findings, which predicted that functional differentiation of the mammary gland conferred some protection. These studies clearly demonstrated that full differentiation in the absence of hormones did not reduce breast cancer risk, and ‘hormonally induced’ differentiation in young parous rodents was needed for breast cancer prevention. 

## 4. Female Hormones, Pregnancy, and Prevention of Breast Cancer 

Early studies, discussed in Section 3, led to the proposal of whether simulating hormonal changes in pregnancy to induce differentiation of the mammary glands as a preventative measure against breast cancer later in life was a feasible and safe preventative strategy. Hence, we propose the controversial question raised by O’Malley and colleagues: can administration of female hormones at a critical stage in reproductive development act as a therapeutic intervention to offer long-term protection against late-onset breast cancer? This poses an interesting challenge, given there is convincing evidence associating estrogen and progesterone in hormone replacement therapy (HRT) with increased breast cancer risk [46] (discussed in Section 6.2). Furthermore, anti-estrogens (tamoxifen) and aromatase inhibitors (blocking estrogen production) are the most common treatment for hormone-related breast cancers [47,48,49]. The identification of molecular changes in the mammary gland of young parous females associated with lifetime protection against breast cancer will provide the knowledge to progress the development of preventative and more efficacious treatments.

### 4.1. Can Estrogen and Progesterone Mimic Pregnancy-Induced Breast Cancer Protection? 

Breast tumors in rodents possess high similarity to ERα-positive and progesterone receptor (PR)-positive human breast tumors. Thus, rodent models have been key in our understanding of breast cancer development [37,43]. As explained by Daniel Medina [50], there are two distinct experimental models of parity/hormone-induced protection. The differences are important in the underlying protective mechanisms and when interpreting the results from these experiments. The “pre-treatment” model implies the administration of carcinogens after the mammary gland has undergone hormonal stimulation, differentiation, and involution. The “post-treatment” model implies exposure to carcinogens and then treatment with hormones for a specified time. In all “pre-treatment” models, full-term pregnancy and combination of estrogen (estradiol-17β) and progesterone conferred protection, leading to a reduction in mammary carcinomas incidence and to a reduction or abrogation of latency. The “post-treatment” model was not as definitive, and the results were more controversial, as will be discussed.

Studies, dating back to 1962, demonstrated that the female hormones estradiol-17β and progesterone administered to rats and mice in doses that mimicked pregnancy reduced the incidence of carcinogenicity and conferred long-term cancer protective effects; however, in these studies neither hormone individually was protective [15,16,17,18]. In contrast, a few studies showed that a critical short-term dose of estrogen alone conferred some protection from mammary carcinogenesis [10,14]. The earliest “post-treatment” experiments by Huggins and colleagues demonstrated 100% of Sprague-Dawley (albino) rats treated with the carcinogen DMBA developed latent mammary tumors [17]. Hormone administration of estradiol-17β and progesterone, after chemical induction of DMBA, reduced latency in all rats and eliminated cancer in approximately 50% of treated rats [17]. Conversely, using the same paradigm, pregnancy was found to accelerate tumor growth, indicating that pregnancy per se was not protective after carcinogen insult. Progesterone had a similar effect as estradiol-17β—just delayed the onset of tumors. Similar experiments in the same rat model suggested that full-term pregnancy had to be completed prior to carcinogenetic exposure to be protective [51]. However, it was later demonstrated that pre-treatment with estradiol-17β and progesterone administered for 21 days in sexually mature female rats also reduced mammary adenocarcinoma incidence [14]. Therefore, in summary, these studies indicated that both full or partial differentiation of the mammary gland reduced breast cancer risk, and this risk could be minimized by short-term exposure to hormone treatment [14]. 

In a study treating rats with estradiol-17β and progesterone for 21 days to mimic the parous protective effects against MNU, a set of 100 markers were identified, displaying persistent changes (upregulated) in the mammary gland compared to virgin rats [52]. One of these changes was RbAp46, a histone-binding protein associated with chromatin remodeling [52]. Other experiments by Yang and colleagues showed that pregnancy and lactation in both in the “pre-treatment” or “post-treatment” model using the MNU carcinogen reduced mammary carcinomas and prolonged latency [53].

In general, the consensus from these studies, and many more not cited, is that short-term administration of estrogen and progesterone, that mimic pregnancy, can reduce malignant tumor formation and breast cancer latency. These changes are accompanied by persistent changes in the cellular and molecular profiles of the mammary cells, making the mammary gland less vulnerable to carcinogens or carcinogenic insults.

### 4.2. Can hCG Mimic Pregnancy-Induced Breast Cancer Protection?

Early studies by Russo and others suggested that human placental hormone chorionic gonadotropin (hCG) provided an inhibitory action, leading to reduction in breast cancer risk, as reviewed in reference [54]. This long-lasting protective effect could also be reproduced by administering placental hCG to rats for 21 days [28]. The mechanism proposed suggested placental hCG was responsible for imprinting the permanent changes in the genomic signature of the mammary gland [54]. These changes occurred within a time window of pregnancy rendering the cells refractory to malignant transformation [54]. Russo’s laboratory compared gene expression profiles of rats exposed to combined estrogen and progesterone or hCG with untreated or pregnant rats [55]. Only the groups with hCG (pregnancy-induced or hCG treatment groups) underwent complete mammary gland differentiation in these studies, although the combination of estrogen and progesterone, as mentioned previously, has been found to have a protective long-term effect against breast cancer. Regulation of genes common to all treated groups, related to cellular differentiation, maintenance of cellular polarity, tight junction formation, and cellular communication, was observed. These experiments further emphasized that permanent changes in the molecular pathways associated with the female hormones (estrogen, progesterone, and hCG), whether associated with partial or full differentiation, provided protection against breast cancer latency. To support the beneficial effects of hCG treatment as a viable alternative to current anti-hormonal therapies, recombinant hCG treatment in post-menopausal women showed a significant decrease in breast cancer cell proliferation [56]. However, it is noted that not all studies supported hCG as a prospective treatment for breast cancer. In contrast, a large prospective study (1191 women with invasive breast cancer and 2257 controls) completed in Finland showed no correlation between early pregnancy hCG serum concentrations and breast cancer risk [57]. What is coming to light is that there are different forms of hCG, which may have opposing effects on breast cancer development, driving different outcomes in breast cancer risk [54]. The differences in hCG isoform expression may, in part, explain the controversial data surrounding hCG and breast cancer management. 

Overall, these studies are illustrative of many other works that explore the influence of the female hormone flux in pregnancy on breast cancer risk. They demonstrate that short-term administration of female hormones, whether it be estrogen and progesterone or hCG to induce mammary gland differentiation, has promise as a therapeutic protective intervention for breast cancer latency. Nevertheless, the challenge is when and how to administer preventative hormones. One of the major questions which still remains unanswered is: what are the molecular changes that govern persistent alterations in hormone-dependent signaling pathways that occur in young parous females and reduce lifetime breast cancer risk? The answer may be in p53’s function, as p53 is believed to be implicated in a number of steps relating to the risk and development of breast cancer.

## 5. p53 in Pregnancy: Cancer Suppressor Protein and Potent Protector again Latent Breast Cancer

### 5.1. p53: Looking beyond the Guardian of the Genome 

For decades, the p53 protein (encoded by the *TP53* gene) has stereotypically been widely researched as the ultimate tumor suppressor protein important in driving cancer cell death through apoptosis or permanent irreversible cell senescence [58,59,60]. It is clear from early and more recent studies that p53 holds a key role in normal cellular programming, differentiation, and survival [61,62,63]. Challenging to p53 importance in normal cellular homeostasis was the observation that that p53-null mice developed no overt defects and were viable, suggesting that p53 was dispensable for normal embryonic development [13,64], albeit a higher percentage of female mice were infertile, and mice were found to be more vulnerable to carcinogens, with increased overall cancer risk [13,64]. The high incidence of cancer in p53-null mice led to the idea that perhaps p53wt was necessary for normal cell differentiation in adult life. On one hand, p53wt maintains a fine balance in normal growth arrest, terminal differentiation, and apoptosis in the undifferentiated, premature adult cell, on the other hand, downregulation of p53wt promotes renewal of stem cell proliferation [61]. In addition, p53-null mice were found to display deregulation of the DNA methylation machinery prior to the increase in tumor formation [65]. In this context, p53 has been shown to act in the surveillance of the epigenetic programming and reprogramming of cells to maintain cell stability [62,66,67].

Today, the actions of p53 extend well beyond its function as the guardian of the genome [68], since it has also been recognized as the guardian of the proteome [69], the guardian of homeostasis [63], the guardian of maternal reproduction [70], and the guardian of cellular respiration and metabolism [71,72,73,74,75,76,77,78,79,80,81]; in addition, it is also a negative regulator of pluripotency and a positive regulator of de-differentiation [82]. The known and ever-increasing emerging roles of p53wt are listed in Table 1.

### 5.2. A Role for p53 in Breast Cancer Origin

Hormonal activation of the p53 pathway at a critical time in mammary development in full-term pregnancy has been identified as one of the most critical determinants of long-term negative or positive alterations in the mammary gland influencing breast cancer risk. This observation was first determined by mimicking early pregnancies in rodents administered defined hormone treatments of estrogen and progesterone which had been shown to have similar protective properties to early full-term pregnancies (described in Section 4.1) [14]. The p53wt status was ascertained under these defined conditions [10]. In support of p53wt as a mediator of hormone-induced resistance to mammary carcinogenesis in early pregnancy, Sivaraman and colleagues demonstrated that estrogen and progesterone induced sustained p53 nuclear expression and, under these conditions, blocked proliferation in response to a carcinogenetic insult in both mice and rats [10]. The p53 protein was functionally active, as demonstrated by an increase in p21/WAF1, a key downstream target of p53. Hence, the translocation of p53 to the nuclear compartment was essential for the hormone-mediated p53-induced cell cycle arrest. The underlying mechanisms for these discrepancies were unclear but believed to be centered on persistent changes in the hormone-induced p53 signaling pathway outcomes [123], therefore leading to the idea that elevated levels of the p53 protein led to permanent changes in the p53 signaling pathways in these mammary cells. These changes provided protection against DNA damage and reduction in breast cancer risk [10,123]. Conversely, late pregnancy allowed for the accumulation of DNA mutations, leading to late-onset cancer and chronic resistance to breast cancer treatment [10]. Further experiments by Medina and Kittrell proposed that p53 upregulation of p21 blocked cells in the G1 phase of the cell cycle and that both p53 and p21 were hormone-regulated [12]. Thus, p53 was the first gene identified as mediating a hormone protective response against breast cancer and latency. These experiments set the precedence, citing p53 as pivotal in the regulation of hormone-induced mammary gland processes. Thus, O’Malley and colleagues proposed a “cell fate” hypothesis for parity-related changes based on their published results, which stated that, at a critical period in adolescence, the hormonal milieu of pregnancy affected the developmental fate of a ‘subset’ of mammary epithelial cells [10,14]. This ‘cell fate’ model hypothesis laid the foundations for the opposing roles of p53 in the protection against breast cancer or in seeding latent breast cancer cells [124].

### 5.3. Hormonal Activation of P19ARF–p53 in Mammary Development 

In support of the role of p53 function in normal mammary development, deletion of the upstream p53 regulator p19 alternative reading frame (p19ARF), the homolog of the human p14ARF protein, led to immortalization of mammary epithelial cells [125]. p19ARF was shown to be regulated during pregnancy by progesterone, and activation of the p19ARF–p53 pathway was necessary for normal proliferation and cell death during mammary gland development in pregnancy. Activation of the p19ARF/p14ARF–p53 pathway has been shown to block cells in both the G1 and G2 phases of the cell cycle, initiating a rapid cell cycle arrest in breast cancer cells [90,98,126]. The cell cycle is dynamic and, depending on the phase of cell cycle arrest (G1, G2, or S phase), different genes We proteins are be switch on or off, hence activating distinct molecular signaling pathways [127]. In speculate that discordance in p53 regulation of the cell cycle pathways could, in part, have negative repercussions in p53-associated differentiation and de-differentiation of breast cells during development.

### 5.4. Lessons from the p53-Null Mammary Gland Transplantations 

A major breakthrough in supporting the role of p53 in governing parity-related changes in the mammary gland came from p53-null mammary gland transplantation studies [13]. By transplanting p53-null mammary tissue into the cleared fat pad of p53wt mice, researchers were able to show a direct correlation between loss of p53 function in the mammary gland and spontaneous development of mammary tumors. Interestingly, the absence of p53 did not alter hormone -responsiveness or normal mammary development in p53-null mammary gland transplants [13]. However, these transplants were more susceptible to tumorigenesis compared to mammary glands harboring p53wt [13]. Exposure of p53null mammary glands to carcinogens (p53null+carcinogen compared to p53null), slightly increased tumor incidence and decreased the latency onset period. The most interesting finding was that exogenous hormone stimulation increased tumor incidence in p53null mammary glands, contrary to the protective effect of hormone stimulation in p53wt mammary tissue. In addition, multiple pregnancies in mice carrying the p53null mammary gland transplants showed no protection against tumor formation. Latent tumors in p53null mice were aneuploid, suggesting that pathogenicity associated with loss of p53 function was not necessarily due to genetic instability. The results from these p53-null transplantation studies are an important milestone in demonstrating the importance of p53 function in parity-related latent breast cancer protection [13].

### 5.5. p53 and Mammary Stem Cell Proliferation

A quote from Beatson’s seminal paper on carcinoma of the mamma (1896) states: “a reasonable ground for thinking the active processes seen in a cancerous tumor are best explained by regarding the epithelium of the part as having taken on the powers of the germinal epithelium” [128]. A likely function proposed for p53 is the control of mammary stem cell renewal [82,129]. In the mammary gland, bipotent mammary stem cells exist [130,131,132]. Progenitor or stem cells possess little proliferation activity and remain dormant until triggered into activity. Potentially, p53 controls the balance between self-renewal, differentiation, and de-differentiation of epithelial progenitor cells. This assertion is based on the observation that deletion of p53 in pluripotent cells showed improvement in the reprogramming efficiency of these cells, and reactivation of p53 initiated cell cycle arrest and stem cell differentiation, as reviewed in reference [82]. Stem cells have been characterized as having the ability to divide asymmetrically, i.e., the two daughter cells produced in cell division possess different proliferative capacity: one daughter retains proliferative capacity, whilst the second daughter cell remains in a quiescent state (progenitor cell). This allows cells to simultaneously generate more stem cells (self-renew) and to produce cells that differentiate [133]. Alternatively, stem cells also use symmetrical division to self-renew, in which both daughter cells have the same fate, i.e., remain in the proliferative state [133]. Stem cell fate is believed to be switched by developmental or environmental factors. Cicalese and colleagues demonstrated that p53 presence controlled the asymmetrical division of self-renewing mammary stem cells: in p53-null mammary glands, stem cells underwent symmetrical division, producing two identical daughter cells, whereas when p53 was present, two daughter cells were produced with different proliferative capacities [134]. This explanation supports a fundamental functional role for p53 in mammary gland differentiation during pregnancy.

### 5.6. p53 and Mammary Cancer Stem Cell Theory

The mammary cancer stem cell theory is based on a model whereby transformation of early progenitor cells or stem cells eventuate in latent tumorigenesis. A central event in the development of cancer is the deregulation of normal stem cell self-renewal, eventually leading to uncontrolled proliferation, aberrant differentiation, and generation of a heterogeneous breast cancer cell population [135,136]. Although this mammary stem cell theory is not proven, there are similarities between the mammary progenitor cell and mammary cancer stem cell phenotypic characteristics, specifically, the plasticity of both cell types, that is, the ability to self-renew or remain in a resting state (dormancy or quiescence), which makes this theory feasible. In general, most breast tumors have lost p53 function, although the majority of post-menopausal breast tumors retain p53wt, making reactivation of p53 a viable target for breast cancer treatments. In light of the findings that p53 is a key player in mammary stem cell regulation, as determined in rodent models, by deduction, dysregulation of p53 function has been cited as a plausible primary origin of latent breast cancer [136].

### 5.7. p53 and Methylation 

Wild-type p53 protein regulates p53 transcriptional programs in the repair of genetic alterations and also by preventing epigenetic abnormalities [66,137]. Tovy and colleagues showed that p53 was important in balancing DNA methylation in embryonic stem cells and in maintaining DNA homeostasis [62]. Cells lacking p53 responded poorly to differentiation signals [62]. These observations were important, given the growing evidence that one of the drivers of cancer is epigenetic aberration. It has been suggested that, under the influence of estrogen and progesterone, p53 participates in chromatin remodeling and initiates epigenetic reprogramming of the parous cell [138]. Epigenetic regulation (DNA methylation, chromatin remodeling, and histone modification) is an important process in development and provides a lasting genetic imprint, restricting gene expression patterns for many years after the modification effector has been removed [139]. As proposed by Ginger and Rosen, these persistent changes may determine mammary cell fate, providing a lasting “memory” and preventing cell lineage anomalies that lead to breast cancer [123]. The p53 gene itself is a prime target for epigenetic alterations. Specific chromatin alterations in the mammary parous glands have been associated with a persistent increase in p53 activity [138]. As mentioned, parous breast cells contain higher levels of heterochromatin compared to breast cells of nulliparous postmenopausal women, which predominantly contain euchromatin. This suggests that, as the nulliparous breast cells did not reach full differentiation, they are more susceptible to transformation and insult from carcinogenic agents. As a consequence, latent breast cancer cells retain the ability to re-acquire the potential to self-renew [28]. The remodeling of the chromatin has been proposed as a decisive step in the protection against breast cancer latency in post-menopausal years [28]. 

## 6. The Connection between p53 Status and Responsiveness to Female Hormones in Breast Cancer

### 6.1. “Paracrine to Autocrine” Hormonal Response in Normal to Breast Cancer Transition

A major difference in the molecular characteristics of normal breast epithelial cells and breast cancer cells is that the majority (approximately 75–85%) of normal breast cells do not express ERα and PR, whereas the majority of breast cancer cells contain high levels of both hormone receptors (ERα+PR+ cells) [140]. Normal breast cells expressing ERα and PR do not self-renew in response to estrogen treatment; these cells send paracrine signals to neighboring cells to proliferate [140]. In contrast, ERα+PR+ breast cancer cells are self-renewing when exposed to estrogen treatment and display an autocrine response. The fundamental switch, or transition, in the molecular response to estrogen provides a survival advantage to breast cancer cells. When and why these changes occur is an enigma. However, it is evident that this switch, or dysregulation of ERα signaling, is most likely to contribute to the early stages of breast cancer development [141]. 

In this section, we review the mounting evidence that p53 and ER activities are mutually regulated in breast cancer. Conceptually, a question worth asking is: is the loss of, or change in, p53 function associated with the signature of “paracrine-to-autocrine” response in the transition from normal to breast cancer cells? In Section 5, we provided an overview of the current thinking about the contribution of p53wt in early pregnancy and protection against latent breast cancer. Here, we summarize the current knowledge on p53 and its perceived role in breast cancer resistance and recurrence. 

### 6.2. Replacement of Female Hormones and Breast Cancer Risk 

This review would be amiss if we did not comment on the seminal findings of the epic Million Women Study by Beral and colleagues, investigating the use of hormone replacement therapy (HRT) in postmenopausal women [46]. In this epidemiological study, the authors investigated the breast cancer risk of using estrogen plus progestin (HRT) compared to estrogen only (HRT). Contrary to the protective effects that estrogen plus progesterone display during pregnancy (Section 4.1), there is, paradoxically, overwhelming evidence to suggest that estrogen plus progestin given to postmenopausal women increase breast cancer incidence [46]. The findings revealed that women given estrogen alone in HRT were comparatively less likely to be diagnosed with breast cancer than women taking HRT containing both progestin and estrogen [142,143,144]. Subsequent studies also revealed that women using HRT were more likely to have increased breast cancer incidence, node-positive, metastatic breast cancers and a higher mortality rate [143,145,146]. These seminal studies exemplify the changing role of hormones: increasing breast cancer risk in postmenopausal women and exerting a protective effect in early pregnancy.

### 6.3. p53 Status in Hormone-Responsive Breast Cancer 

The p53 tumor suppressor is intimately associated with inhibition of cancer and, in general, mutations in the *TP53* gene that disrupt cell cycle inhibition are associated with the onset of more than 80% of all cancers [147,148,149]. Nevertheless, somatic mutations and deletions only occur in 20–25% of breast cancers [150]. This observation suggests that mutations in the *TP53* gene are not the main culprits in breast cancer development and progression. The majority of breast cancers occurring later in life (in postmenopausal women) are hormone-responsive (ERα-positive) and, interestingly, harbor functional wild-type *TP53* [5,151]. In many breast cancers, the p53 protein is continuously degraded by the process of ubiquitination, and, therefore, p53 is non-functional [152,153]. Restoring p53 function by reactivation of p53 through therapeutic intervention, including radiation, chemotherapy, or mimetics of p14ARF, rely mainly on p53 promoting cancer cell death [58,153,154,155,156,157]. 

As mentioned in Section 6.1, the “paracrine-to-autocrine” hormonal response is a major change in the “normal-to-breast-cancer” transition; hence, breast cancer cells proliferate in response to estrogen. ERα positivity is used as a diagnosis tool for designing breast cancer treatment strategies and predicting prognosis. Therefore, in ERα-positive breast cancers, anti-estrogens, such as tamoxifen, are primary therapies used in treatment and prevention. The most pressing problems with hormonal therapies are resistance and recurrence. Despite an apparent initial success of treatment, many ERα-positive breast cancers have intrinsic or acquired resistance to endocrine therapy [48,158,159]. Latent recurrence is prevalent, particularly in ERα-positive breast cancers, and is associated with cell dormancy, rather than with death, after treatment, as reviewed in reference. Recurrence usually manifests as metastatic cancer and can occur months or decades after the initial therapy. These metastatic breast cancers are more difficult to treat and have higher mortality rates. The mechanisms underlying breast cancer recurrence and metastasis and how we can intervene in these processes are the major challenges faced by breast cancer researchers. 

### 6.4. Molecular Basis for p53 and ERα Association in Resistance and Recurrence in Breast Cancer

Although reactivation of the p53 signaling pathway has been considered as a good breast cancer treatment strategy, there is growing evidence at the molecular level to suggest an association between ERα and p53, which protects breast cancer cells from dying [90,93,98,160,161,162,163]. Studies by Konduri and colleagues, exploring the role of p53 in breast cancer, found that ligand-bound ERα physically stabilized p53 and prevented p53 degradation [93,98,160,162,163,164]. Furthermore, this interaction stopped the accumulation of DNA damage in breast cancer cells and strongly supported the idea that the binding of p53 to ERα–estrogen protects breast cancer cells from apoptosis, hence providing a base for the resistant phenotype. Liu et al. reported that ERα altered p53’s function via direct binding, and this suppression was achieved by the activation of the ERα function-2 domain and the C-terminal regulatory domain of p53 [162]. ERα has also been shown to bind to p53 and inhibit its transcriptional repression of anti-apoptotic genes, thus contributing to the ERα anti-apoptotic function in ERα-positive breast cancer cells [163]. ERα-positive breast cancers with p53wt were more likely to be responsive to anti-estrogens than ERα-positive breast cancer with mutated p53 [160]. This response was due not only to the inhibition of estrogen binding to ERα and of ERα-dependent transcription, but also to the reactivation of p53wt by disrupting the p53–ERα complex [160]. p53 activation by estrogen in postmenopausal breast cancer has been associated with prevention of apoptosis [165], and high levels of estrogen in ERα-positive breast cancers have been shown to correlate with high levels of p53 [166]. Recently, Berger et al. showed that estrogen binding to ERα enhanced p53 activation, whereas anti-estrogens reduced p53 activation [167]. 

Our laboratory has demonstrated that activation of the p53 pathway in ERα-positive breast cancer cells rapidly, within hours, shuts down DNA replication, downregulates proteins involved in apoptosis, and increases the levels of proteins that are involved in normal breast cell metabolism [90,97,98]. Other studies in the Myles Brown laboratory used a genome-wide approach to understand the underlying mechanism by which ERα protects breast cancer cells from the p53 apoptotic function and identified the modulation of a specific subset of p53 and ERα target genes [93]. These studies question the canonical role of p53 as a tumor suppressor in normal breast physiology. 

### 6.5. p53 and ERβ Association in Breast Cancer Prevention

In 1997, a second ER was identified, named *ER*β [168,169]. The two receptors are transcribed from different chromosomes; however, they have high homology in their DNA-binding and carboxyl-terminal ligand-binding domains. Both receptors bind estrogen (17β-estradiol), albeit with different binding affinities and responsiveness to selective estrogen receptor modulators (SERMs), such as tamoxifen and raloxifene [170]. The major differences between the two receptors lie in their amino-terminal domains, which bear little similarity [171]. Each of the ER transcripts have different downstream functions, as identified in ER*α* and ERβ knockout (KO) mouse models [172]. In selective ER*α* and ERβ KO mouse models, ER*α* was shown to be essential for normal reproductive development [173]. In contrast, although ER*β*-KO mice had decreased side branching and partial alveolar development, the mammary glands secreted milk normally, and mothers were able to nurse their young [173]. Unlike ERα, which is associated with breast cancer proliferation, ERβ has an opposite effect in hormone-dependent cancers and is associated with an inhibitory role in tumorigenesis and metastasis [174]. Conventional ERβ-KO mice alone did not lead to tumorigenesis [175]. Alternatively, in conditional ERβ and p53 KO mice, Bado and colleagues demonstrated that a concomitant loss of ERβ and p53 induced early onset of basal-like mammary tumors [176]. In two recent independent publications, ERβ was shown to interact with p53, reduce p53–ERα binding affinity, and antagonize transcription regulation by p53–ERα [177,178]. Thus, if there is competitive binding between the ER proteins with p53, it has been suggested that the ratio of ERβ and ERα may affect estrogen responsiveness in breast cancer [178]. The pro-apoptosis response of ERβ in breast cancer cells also involved epigenetic changes in the methylation of histones affecting gene regulation and, hence, cellular activities of the cells [177]. Thus, these studies elucidated a novel anti-proliferative and pro-apoptotic mechanism in breast cancer involving the ERβ–p53 interaction. 

In essence, the role of p53 as a protector or perpetrator in breast cancer may very well be dependent on the ratio of ERα and ERβ in breast cancer. In turn, the crosstalk between ERα, ERβ, and p53 has the potential to affect hormone-dependent breast cancers responsiveness to endocrine and other breast cancer therapies [177,178]. 

### 6.6. Hypothesis of p53 Activation Changing Metabolism and Breast Cancer Survival 

Beatson’s theory from his doctorate dissertation was that lactation was very close to being a cancerous process and, if arrested in its progression, could become carcinogenic, as cited in reference [179]. In other words, a breast cancer cell is a normal mammary cell “lost in translation” during the re-programming process in pregnancy.

MCF-7 cells are derived from the luminal breast epithelial, which consists of cells that differentiate to become milk-producing cells. This cell line has provided vital information on hormone regulation of breast cancer and treatments to combat this disease. We have recently demonstrated that activation of p53 in MCF-7 breast epithelial cells initiated changes in cell morphology and cellular metabolism consistent with a differentiated phenotype and was most likely to be important in cell survival and recurrence in ERα-positive breast cancer cells [90,97,98]. The potential functional role of p53 in breast cancer metabolism is reviewed in our recent article [180]. We used global proteomic labeling to determine changes occurring in ERα-positive breast cancer cells after p53 activation. As part of the global protein changes, we described a unique snapshot profile analysis of the differential regulation of the annexin and S100A calcium-binding-associated protein family [97]. The annexin family comprises proteins that are important regulators of normal cellular function, and tight regulation of calcium is critical in breast homeostasis during pregnancy and lactation, the primary function of the breast [181]. A tight reprogramming of calcium regulators during pregnancy is vital to avoid aberrant cell proliferation and death during the lactation process. Alternatively, aberrations in calcium regulation are key features of breast cancer [182,183]. Aberrant expression of individual annexins and S100A proteins has been associated with malignant transformation [184,185,186,187,188], tumor invasion [189,190,191], metastasis, angiogenesis, and drug resistance [192,193,194], depending on breast cancer sub-type. The hypothesis resulting from these studies is that, although MCF-7 is a cancer cell line, it has a functional memory of self, that is, of lactation. Understanding the changes that occur after the reactivation of p53 in these cells in association with hormone exposure should provide useful clues to defining the role of p53 in the development of breast cancer, and help gain insights into the process leading to breast cancer recurrence. 

### 6.7. Problems Raised with p53-Based Treatments in ERα-positive Breast Cancer

Our studies and the work of other groups have strongly suggested that re-activation of the p53 signaling pathway is strongly implicated in chemotherapy- and endocrine therapy-resistant breast cancers, especially in the most prevalent breast cancer sub-type (ERα+/p53+) [93,98]. Activation of the p53 pathway in ERα-positive breast cancer cells rapidly induces cell cycle arrest; however, the cells remain viable, show changes in morphology and metabolic functions (increase in mitochondria biomass and readout function), can remain in a dormant state, and have the potential to reproduce by endoreplication in cell culture [90,98]. Although the activation of the p53 axis has been shown to rapidly stop cell division, this treatment does not necessarily lead to cell death [90,97,98]. More recently, a role of p53 in energy metabolism and survival has been proposed, providing cancer cells with a survival advantage; however, the mechanisms are less well understood [77,99,195]. Therefore, understanding the basic metabolic processes that lead to chemo- and endocrine resistance and relapse in breast cancer and that are based on the activation of the p53 tumor suppressor survival pathway is of high importance.

The theory that the p53 protein suppresses cell cycle progression, induces resistance to apoptosis, and maintains the breast cancer cells viable through metabolic reprogramming [90,98] is an important concept underlying breast cancer endocrine resistance and recurrence and needs to be explored in more detail.

## 7. Summary and Conclusions

**Figure 1:**
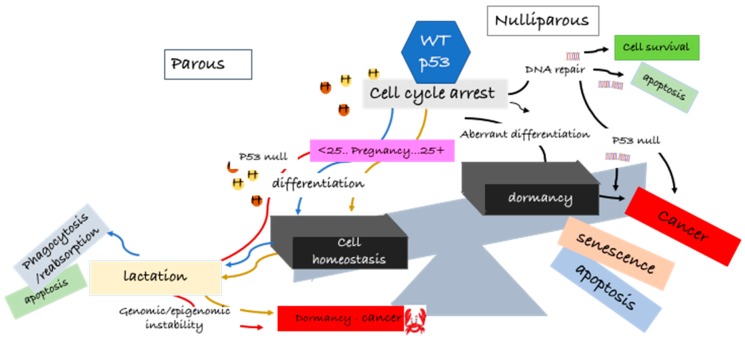
A role for p53wt in breast cancer origin and latency—p53wt “good guy–bad guy” hypothesis. During pregnancy (before 25 years of age), under the influence of the female hormones (estrogen and progesterone), p53 participates in stepwise chromatin remodeling and epigenetic reprogramming. These reprogramming events imprint a lasting protective signature on mammary cells, maintaining homeostasis (blue line). After lactation, wasted cells are phagocytosed and reabsorbed or undergo apoptosis. Mammary glands in females over the age of 25 years undergo the same process; however, because of the potential genomic and epigenetic instability, resulting from the continuous pre-pregnancy hormonal flux over the years, the mammary cells are not protected against latent breast cancer. The mammary cells may remain in a pre-cancerous dormant state for decades until stimulated by mitogens to proliferate (gold line). In nulliparous females, p53null mammary cells also produce active lactating cells but are highly vulnerable to spontaneous cancer (red line). p53 tries to repair the damaged cells for survival (black line). If DNA damage cannot be repaired, cells undergo apoptosis, senescence, or remain in a pre-cancerous dormant state (black line). In aberrant differentiation, 53wt tries to repair the DNA mismatches. If unable to complete the repair, the cells undergo apoptosis or become precancerous cells (black lines). Aberrant differentiation can also lead to dormancy and, thereafter, apoptosis, senescence, or emerging of latent breast cancer cells (black line). Hypothetically, recurrence of breast cancer may follow the process of dormancy, i.e., cells do not die but remain in a vulnerable pre-cancerous state.

In summary, we do not know the extent of the function of p53 in the origin of latent hormone-responsive breast cancers, and this review does not delve into the many complexities of the normal p53 function. What is emerging from these cited reports and others is that p53 expression at a critical timing in young age pregnancy is pivotal in preventing mammary gland tumorigenesis. The underlying associated molecular changes could be permanent reprogramming of cellular events and/or DNA methylation, imprinting a protective signature and maintaining normal cell homeostasis. With older age pregnancies or in nulliparous women, accumulation of DNA mutations or other aberrations in the mammary gland abrogate this protective signature, and the cells are more susceptible to carcinogenic agents. Hormonal activation of the p53 in the mammary gland is implicated in the development of latent breast cancer. Confidence in the protective effect of p53 comes from p53null rodents, where, in the absence of p53, spontaneous tumors are found in the mammary glands, independent of age, as summarized in Figure 1.

How can we utilize this knowledge for more efficacious treatments and to prevent recurrence and metastasis? While the tumor suppressor role of p53 in breast cancer treatment is well recognized, the evidence supporting an opposite action of p53 in treatment resistance and recurrence in breast cancer is not as clear. Breast cancer recurrence may well be a consequence of the heterogeneous nature of the initial tumor, confounded by the change in hormone signaling from a paracrine to an autocrine proliferative response upon estrogen exposure. Breast cancer cells have a “memory of self”, and investigations in hormone-responsive breast cancer cells after p53 activation show characteristics of aberrant mammary differentiation, followed by dormancy, mediated by p53 activation. Further studies into how p53 protects breast cancer cells from cell death by switching cellular metabolism, downregulating apoptotic proteins, and initiating a cellular differentiation phenotype reminiscent of the normal mammary function is key to our understanding of recurrence in latent breast cancer development and a basis for the design of more efficacious treatments. 

We need to look to the past to move on to the future and we now have the technology to do so.

## Figures and Tables

**Table 1:** Known functions of wild-type p53.

Function	Summary—Key Regulatory Functions	Reference
Homeostasis regulators	p53 is a key regulator of replication homeostasis within a DNA restart network and is essential for DNA methylation homeostasis in stem cells. It also plays a key role in the regulation of metabolic homeostasis. The function of p53 in cellular energy homeostasis and metabolism is emerging as a critical factor for tumor suppression.	[62,72,83,84,85]
Cell cycle arrest	One of the best-understood function of p53 is to promote cell cycle arrest. Cell cycle arrest by p53 is mainly mediated by the transcriptional activation of p21/WAF1 and is reversible after downregulation of p53.	[72,83,86,87,88,89,90]
Apoptosis	It has been confirmed in many studies that induction of apoptotic death in nascent neoplastic cells is the principal mechanism by which p53 suppresses tumor development. p53 induces apoptosis in nontransformed cells mostly by direct transcriptional activation of the pro-apoptotic BH3-only proteins PUMA and (to a lesser extent) NOXA.	[86,87,91,92,93]
Cellular senescence	Chronic p53 activation can result in senescence of tumor cells. Senescent cells have unique features, such as large cell size, active autophagy, high lysosomal SA-b-gal activity, and secretion of proinflammatory cytokines. Senescence is a unique state of cell cycle arrest that is highly stable but is not completely irreversible. Through the induction of senescence, p53 promotes and achieves permanent inhibition of cell proliferation.	[86,87,93,94,95,96,97,98,99]
Cellular quiescence	p53 is activated during both quiescence and senescence. Evidence suggests that p53 activation contributes to the quiescent growth arrest and is a reversible process.	[100,101,102].
Proliferation/survival	There is a strong direct correlation between accumulation of p53 protein and tumor proliferation rate. Expression of mutant p53 protein was associated with high tumor proliferation rate, early recurrence, and death in breast cancer. Recently, it was noted that p53 can also contribute to cell survival.	[86,90,91,99,103,104]
Autophagy	In most cases, p53 positively regulates autophagy in tumor cells by inhibiting mTOR pathways via the activation of AMPK. p53 also promotes autophagy by inducing various autophagy-related genes. Autophagy is considered a tumor suppressive mechanism that removes unfolded proteins, damaged cellular components, and damaged organelles to maintain cellular homeostasis.	[96,105,106,107,108,109,110,111]
Metabolism	p53 promotes oxidative phosphorylation and dampens glycolysis in cells; disruption of this balance is associated with mutations in p53 and oncogenic transformation. P53 plays a role in alterations seen in glycolysis, gluconeogenesis, and aerobic respiration. Altered metabolism can contribute to malignant transformation, and cancer cells become dependent on these changes. p53 regulates various metabolic pathways, helping to balance glycolysis and oxidative phosphorylation, limiting the production of reactive oxygen species, and contributing to the ability of cells to adapt to and survive mild metabolic stresses.	[72,77,84,87,88,99,112,113]
DNA repair	p53 plays a prominent role as a facilitator of DNA repair by halting the cell cycle to allow time for the repair machinery to restore genomic stability; for example, p53 coordinates DNA base excision repair in the cells, and this mechanism is impaired in p53-inactivated cells. Within a DNA restart network, p53 functions as a keystone regulator in DNA replication homeostasis.	[85,114]
Oncogenic functions	p53wt is a tumor suppressor gene; mutations in this gene promote oncogenic capacity. Thus, mutant p53 is an actionable target of clinical antitumor therapies. p53 loss of heterozygosity (LOH ) is a critical prerequisite for missense mutant p53 stabilization and gain of function in vivo.	[92,115,116,117]
Epigenomic regulator	p53 is not only a pivotal guardian of genomic stability, but also an epigenetic regulator. Epigenomic regulation is a new function of p53, contributing to its tumor suppressor activity. It is thought that the ability of p53 to maintain DNA methylation balance is an important contributor to its tumor suppressor capacity and that loss of p53 may result in cancer initiation by increasing cellular heterogeneity and epigenetic promiscuity.	[62,93,104,118,119]
Regulating multiple tumor suppressor genes	Under normal low-stress conditions, p53wt is capable of maintaining the expression of a group of important tumor suppressor genes at baseline, which could contribute to p53-mediated tumor suppression. p53 mutations, with inactivation of multiple tumor suppressor genes in parallel, could lead to the high frequency of p53 mutations in cancer.	[120]
Mutant p53 functions	Unidentified mechanisms by which mutp53 confers oncogenic functions by promoting cancer cell adaptation to metabolic stresses.	[88,92,121]
Non-canonical cell death	Transcriptional regulation of downstream targets: caspase-independent apoptosis, autophagy, ferroptosis, mitotic catastrophe, paratosis, pyrotosis, efferocytosis (clearing dead cell debris).	[122]
